# Correlation between disease activity and serum ferritin in clinically amyopathic dermatomyositis with rapidly-progressive interstitial lung disease: a case report

**DOI:** 10.1186/s13104-018-3146-7

**Published:** 2018-01-16

**Authors:** Kazuhiro Yamada, Kazuhisa Asai, Atsuko Okamoto, Tetsuya Watanabe, Hiroshi Kanazawa, Mai Ohata, Masahiko Ohsawa, Kazuto Hirata

**Affiliations:** 1 Department of Respiratory Medicine, Graduate School of Medicine, Osaka City University, 1-4-3, Asahi-machi, Abeno-ku, Osaka, 545-8585 Japan; 2 Diagnostic Pathology, Graduate School of Medicine, Osaka City University, 1-4-3, Asahi-machi, Abeno-ku, Osaka, 545-8585 Japan

**Keywords:** Clinically amyopathic dermatomyositis, CADM, Rapidly progressive interstitial lung disease, RP-ILD, Melanoma Differentiation-Associated gene 5, MDA5, Corticosteroids, Tacrolimus, Cyclophosphamide

## Abstract

**Background:**

Clinically amyopathic dermatomyositis with anti-Melanoma Differentiation-Associated gene 5 (MDA5) antibody often presents with severe interstitial lung disease. Although serum ferritin level is known to reflect interstitial lung disease activity, there are few case reports describing this association.

**Case presentation:**

A 58-year-old man was referred to our outpatient clinic with a 3-week history of cough and respiratory distress. He had erythema over the V area of the neck and a Gottron’s sign. Chest computed tomography revealed diffuse ground-glass opacities and reticular shadows in both lungs. Test for anti-MDA5 antibody was positive. After admission, he received triple combination therapy (methylprednisolone pulse therapy, tacrolimus, and cyclophosphamide). However, his respiratory condition worsened as the serum ferritin level increased. Despite no apparent deterioration on chest radiography, he ultimately died due to respiratory failure.

**Conclusions:**

In this case, triple combination therapy was not effective for the patient’s respiratory condition. The serum ferritin level was correlated with disease activity and was more useful than chest radiography for monitoring clinical status.

## Background

Patients with clinically amyopathic dermatomyositis (CADM) with anti-Melanoma Differentiation-Associated gene 5 (MDA5) antibody often develop treatment-resistant, and rapidly-progressive interstitial lung disease (RP-ILD) [1]. In CADM-ILD, 80% of fatal cases died due to refractory ILD within 90 days of initial presentation [2]. Early treatment of CADM with RP-ILD is essential to improve the prognosis [3, 4]. Triple combination therapy (corticosteroids, tacrolimus, and cyclophosphamide) is considered effective for CADM with RP-ILD. Although the serum ferritin level is known to predict prognosis in anti-MDA5 antibody-associated ILD with dermatomyositis and is also known to reflect disease activity, few case reports have described this association. We report a case in which the serum ferritin level was closely correlated with disease activity.

## Case presentation

**Fig. 1:**
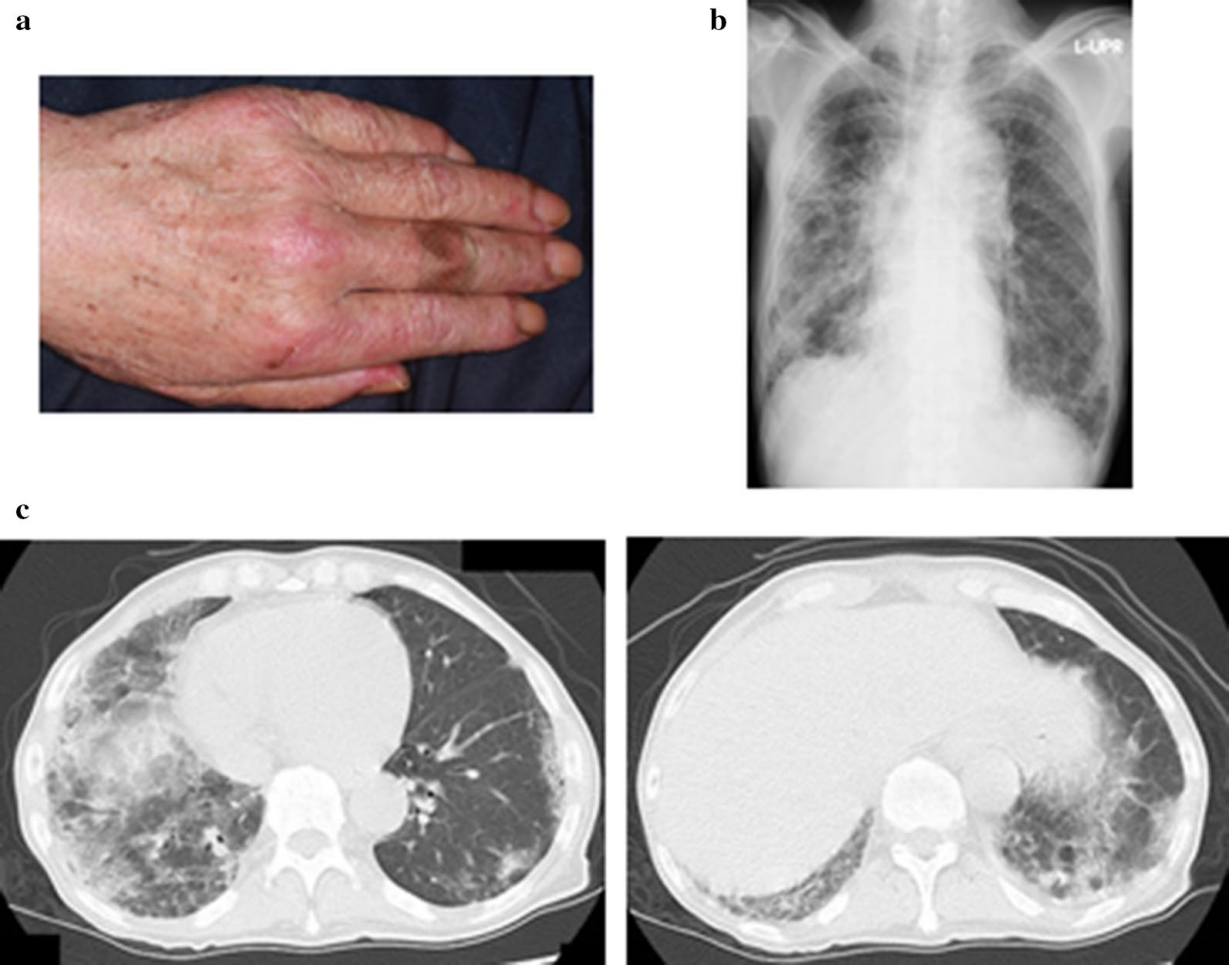
**a** The clinical presentation on admission. Papules were observed near the interphalangeal articulations (Gottron’s sign). **b** Chest radiography showed bilateral reticular shadows, in the right lung. **c** Chest CT scan showed bilateral, asymmetric, ground-glass opacities and reticular shadows, predominantly in the lower lung

**Fig. 2:**
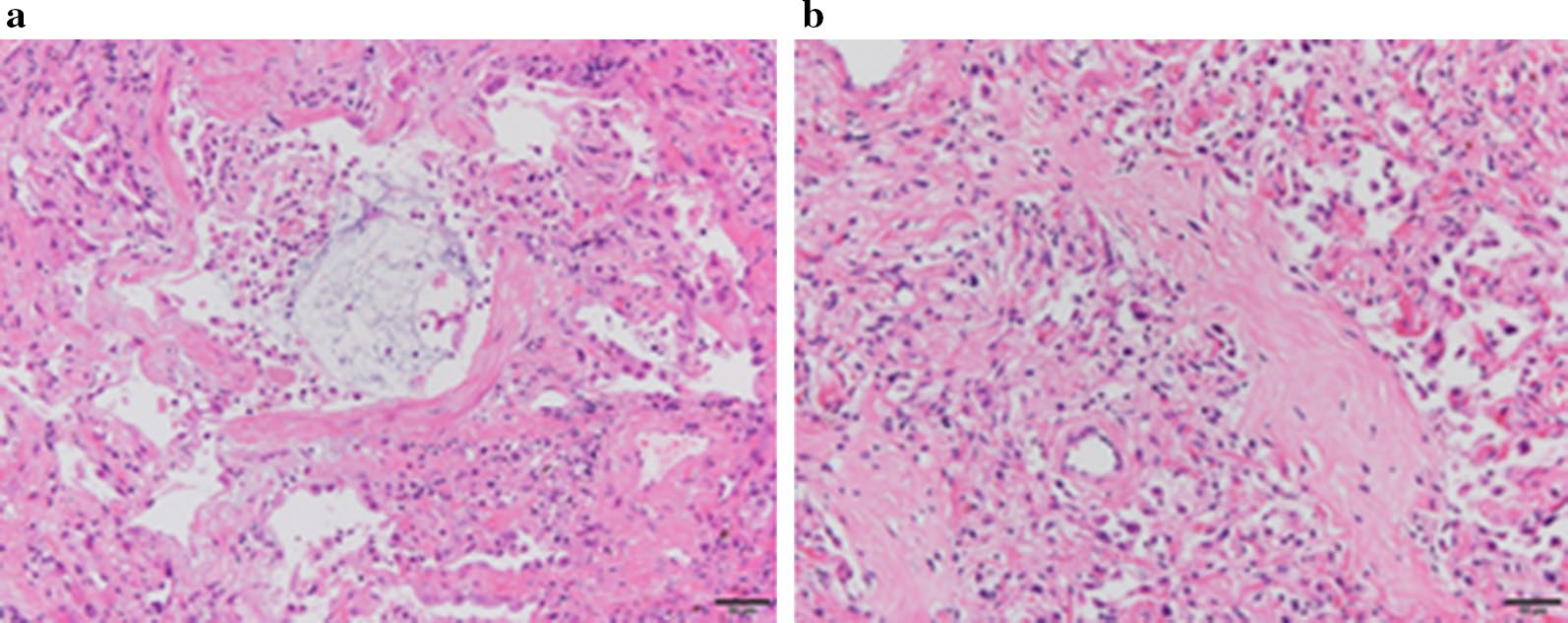
**a** Transbronchial lung biopsy showed proliferative-phase diffuse alveolar damage with a glassy eosinophilic substance in the alveolar spaces and interstitial fibrosis associated with type 2 pneumocyte hyperplasia. **b** Autopsy lung section showed fibrotic-phase diffuse alveolar damage with diffuse fibrotic change and type 2 pneumocyte hyperplasia, with lymphocyte infiltration into alveolar septa

**Fig. 3:**
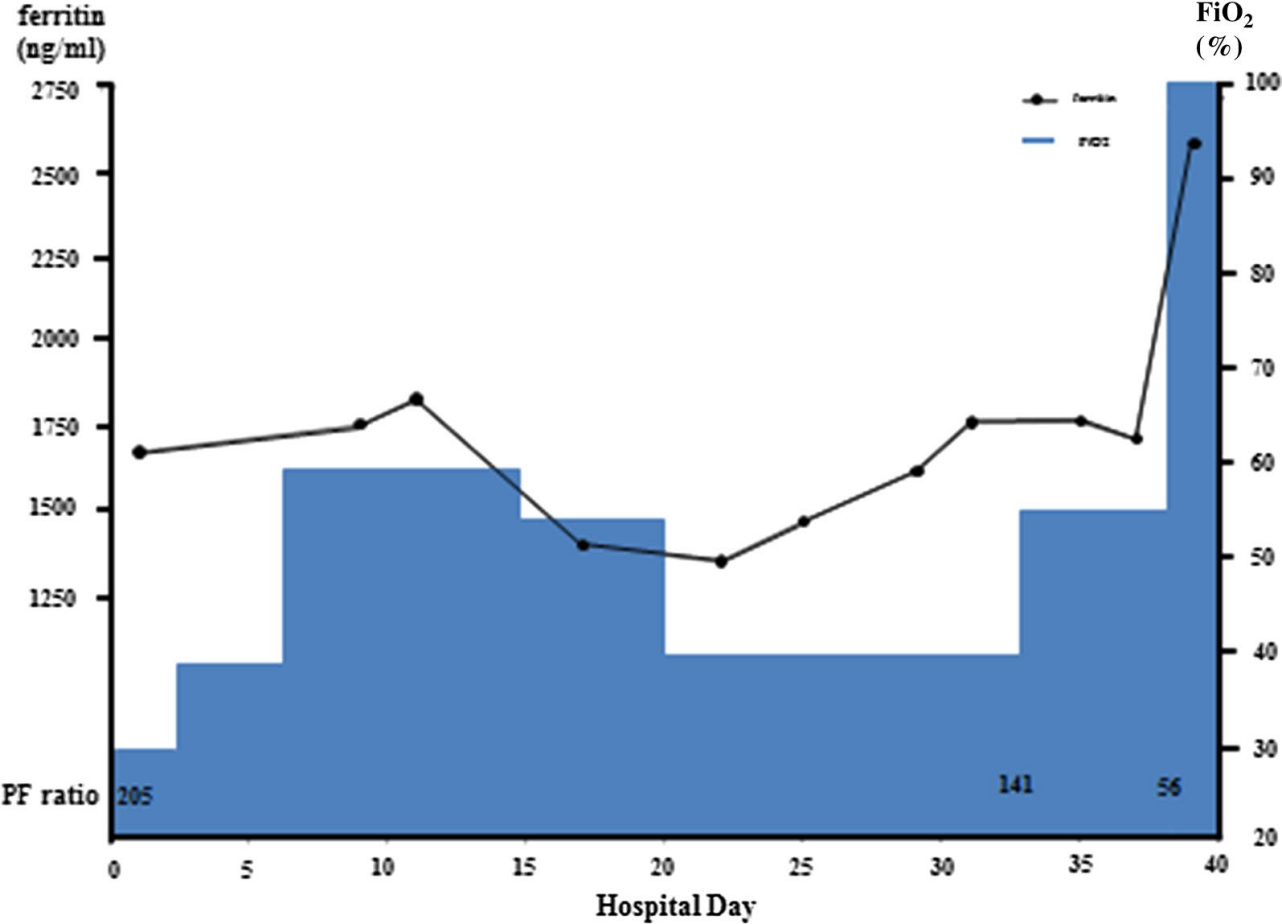
The concentration of oxygen required changed in association with the serum ferritin level

**Fig. 4:**
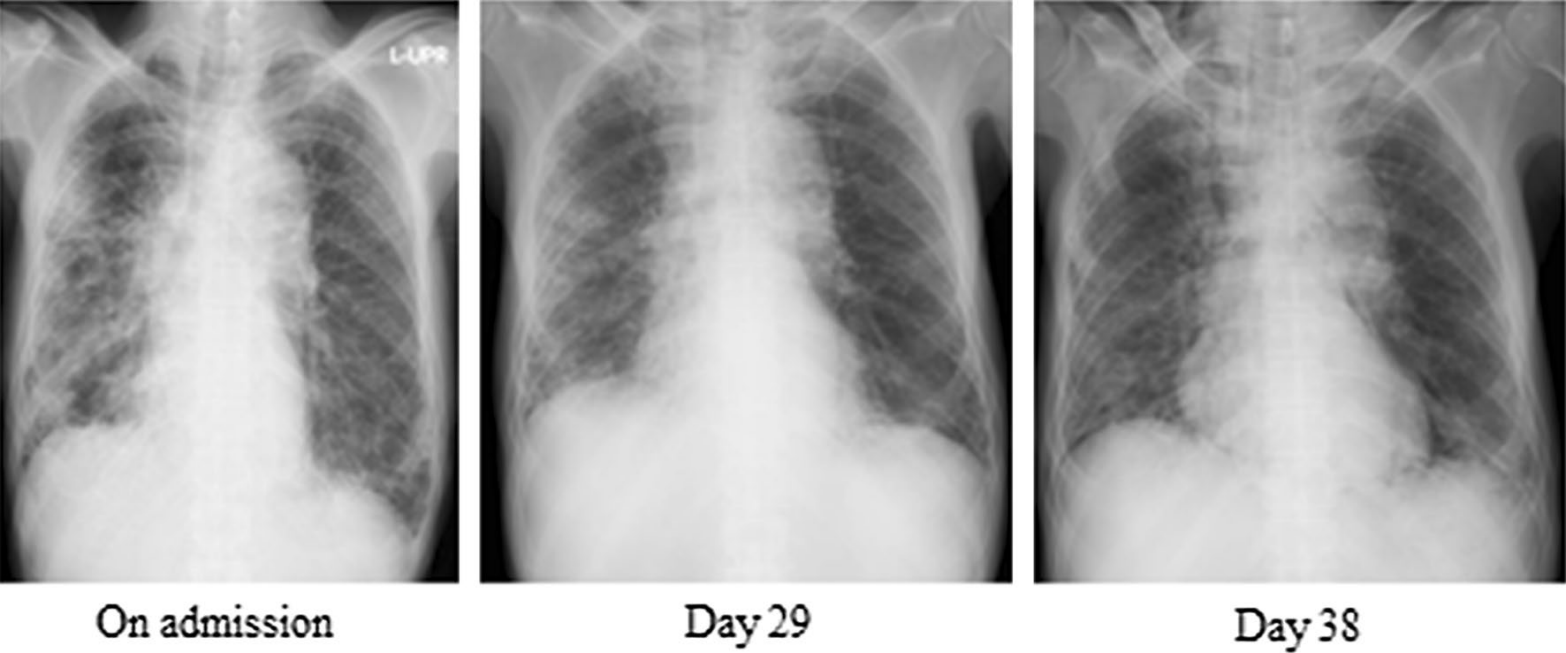
Chest radiography did not show apparent deterioration in association with the serum ferritin level

A 58-year-old man was referred to our outpatient clinic with a 3-week history of cough and respiratory distress. He was a current smoker (40 pack-years) but had no other medical history. His body temperature was 37.2 °C, blood pressure was 82/51 mmHg, pulse rate was 110 beats/min, respiratory rate was 20 breaths/min, and oxygen saturation was 84% in room air. He had erythema over the V area of the neck and a Gottron’s sign (Fig. 1a), but no signs of muscle weakness. Fine crackles were auscultated in the bilateral lung bases. The serum level of creatine kinase was not elevated (68.0 U/L). The serum aldolase, Krebs von den Lungen-6, lactate dehydrogenase, and ferritin levels were increased (11.0, 1.040, 658 U/L and 1.679 ng/mL, respectively). Anti-aminoacyl tRNA synthetase and other antibodies suggestive of autoimmune disorders were not detected (Table 1). Chest radiography showed bilateral reticular shadows, predominantly in the right lung (Fig. 1b). Chest computed tomography (CT) showed bilateral, asymmetric, ground-glass opacities and reticular shadows, predominantly in the lower lungs, suspicious for CADM with RP-ILD (Fig. 1c). Bronchoalveolar lavage was performed from right B5. The total cell count was 0.394 × 10^5^/mL, and the percentages of neutrophils, eosinophils, and lymphocytes were 47.2, 1.8, and 21.6%, respectively. A transbronchial lung biopsy specimen from right B2b showed a diffuse alveolar damage pattern (Fig. 2a). Following bronchoscopy, methylprednisolone pulse therapy (1000 mg daily for 3 days) and tacrolimus were initiated, followed by cyclophosphamide pulse therapy (500 mg for 1 day). After starting treatment, test for anti-MDA5 antibody was confirmed to be positive. Therefore, the diagnosis was CADM with anti-MDA5 antibody. His respiratory condition gradually improved, and the serum ferritin, lactate dehydrogenase, and aldolase levels decreased. However, after 4 weeks, his respiratory condition gradually worsened in association with an increasing serum ferritin level, but there was no deterioration on chest radiography (Figs. 3, 4). Although courses of methylprednisolone and cyclophosphamide pulse therapy were repeated, his respiratory condition worsened, and he died on hospital day 43. An autopsy showed diffuse fibrosis in all three right lung lobes. Some lymphocytic infiltrate was present in the alveolar wall. Diffuse fibrosis was present and local organizing pneumonia was observed in the left lung (Fig. 2b).

**Table 1:** Laboratory data

Hematology		Serology	
RBC	467×10^4^/μL	CRP	3.28 mg/dL
Hb	13.4 g/dL	IgG	1742 mg/dL
Ht	40.7%	IgA	443 mg/dL
WBC	9400/μL	IgM	68 mg/dL
Neutro	76%	KL-6	1040 U/mL
Lymph	14%	Immunological test	
Mono	4%	RF	< 5 U/mL
Eosino	5%	Anti nuclear Ab	40-fold
Plt	37.8 × 10^4^/μL	Anti CCP Ab	< 0.6 U/mL
Blood chemistry		Anti Ds-DNA Ab	2.9 U/mL
TP	7.0 g/dL	Anti RNP Ab	Negative
Alb	2.7 g/dL	Anti SS-A Ab	< 1.0 U/mL
T-bil	0.5 mg/dL	Anti SS-B Ab	< 1.0 U/mL
AST	72 U/L	Anti Scl-70	< 1.0 U/mL
ALT	27 U/L	PR3-ANCA	< 1.0 U/mL
LDH	658 U/L	MPO-ANCA	< 1.0 U/mL
CK	68 U/L	Anti ARS Ab	< 5.0 U/mL
BUN	21 mg/dL	Blood gas (O_2_ 3 L/min by nasal plugs)	
Cre	0.65 mg/dL	PH	7.444
		PaCO_2_	36 Torr
		PaO_2_	72 Torr
		HCO_3_^−^	24.3 mEq/L
		B.E.	1.0 mEq/L
		SaO_2_	94%

## Discussion and conclusion

Reportedly increasing in the past decade, CADM-ILD is often refractory and rapidly progressive, and CADM-ILD with anti-MDA5 antibody has a poor prognosis. For early initiation of proper treatment, quick and accurate diagnosis is needed. This case showed a Gottron’s and V-neck signs; however, muscle weakness was not apparent. Chest CT showed bilateral, asymmetric, ground-glass opacities and reticular shadows, in the lower lungs, suspicious for CADM-ILD. Testing for anti-MDA5 antibody was positive. For initiation of proper treatment, accurate estimation of prognosis is needed. Gono et al. reported that elevated serum ferritin levels were related to the severity of ILD in patients with dermatomyositis. Interestingly, they found that the cumulative survival rate was lower in the subset with ferritin ≥ 1500 ng/mL than in the subset with ferritin < 1500 ng/mL [5]. Ferritin is the primary iron storage molecule; it is secreted by activated macrophages and plays a crucial role in sequestration of potentially harmful reactive iron molecules. High serum ferritin level may reflect aberrant activation of macrophages in patients with CADM-ILD. In this case, the serum ferritin level was 1679 ng/mL on the day of admission. A transbronchial lung biopsy specimen from right B2b showed proliferative phase diffuse alveolar damage (DAD). DAD is reportedly associated with a poorer prognosis than other histopathological patterns, such as unusual interstitial pneumonia [6].

Based both on the serum ferritin level and pathological findings, we predicted a poor prognosis and immediately initiated intensive treatment with triple combination therapy (corticosteroid, tacrolimus, and cyclophosphamide). T cells play a vital role in development of ILD in polymyositis/dermatomyositis [7, 8]; therefore, use of calcineurin inhibitors of T-cell activity is considered important. Cyclosporine inhibits the activity of calcineurin by binding to cyclophilin, whereas tacrolimus exhibits inhibitory activity by binding to FK binding protein. The pharmacological effect of tacrolimus is 100 times stronger than that of cyclosporine, and its half-life is longer than that of cyclosporine [9]. The superiority of tacrolimus to cyclosporine in renal, liver, or bone marrow transplantation has been demonstrated in randomized controlled trials [10–13]. Kameda et al. reported that 5 of 10 dermatomyositis patients with RP-ILD responded to triple combination therapy [14]. These reports indicate that triple combination therapy is warranted in cases of CADM with RP-ILD. Therefore, we administered cyclophosphamide from the beginning of treatment. Unfortunately, triple combination therapy was not effective. The patient’s respiratory condition worsened without apparent deterioration on chest radiography. An adequate treatment-monitoring tool is needed to enable disease control.

The serum ferritin level is reportedly correlated with disease activity in anti-MDA5 antibody-associated ILD with dermatomyositis [5, 15] In this case, the treatment was initially effective; however, his respiratory condition worsened in association with an increasing serum ferritin level, and he died on hospital day 43. Lung specimens at autopsy showed fibrotic phase DAD. The serum ferritin level was closely correlated with disease activity (Fig. 3). There was no apparent deterioration of ILD on chest radiography (Fig. 4). This case indicated that a high serum ferritin level in anti-MDA5 antibody-associated ILD with dermatomyositis was very useful for monitoring disease activity. High serum ferritin level reflected disease activity in anti-MDA5 antibody-associated ILD with dermatomyositis. In this case, the serum ferritin level was more useful than chest radiography for monitoring of disease progression.

## Abbreviations

CADM: clinically amyopathic dermatomyositis
MDA5: Melanoma Differentiation-Associated gene 5
RP-ILD: rapidly progressive interstitial lung disease
CT: chest computed tomography
DAD: diffuse alveolar damage
